# Analysis of safety and efficacy of proton radiotherapy for IDH-mutated glioma WHO grade 2 and 3

**DOI:** 10.1007/s11060-022-04217-y

**Published:** 2023-01-04

**Authors:** Tanja Eichkorn, Jonathan W. Lischalk, Juliane Hörner-Rieber, Maximilian Deng, Eva Meixner, Anna Krämer, Philipp Hoegen, Elisabetta Sandrini, Sebastian Regnery, Thomas Held, Semi Harrabi, Christine Jungk, Klaus Herfarth, Jürgen Debus, Laila König

**Affiliations:** 1 Department of Radiation Oncology, Heidelberg University Hospital, Im Neuenheimer Feld 400, 69120 Heidelberg, Germany; 2 Heidelberg Institute of Radiation Oncology (HIRO), Heidelberg, Germany; 3 National Center for Tumor Diseases (NCT), Heidelberg, Germany; 4 Heidelberg Ion-Beam Therapy Center (HIT), Department of Radiation Oncology, Heidelberg University Hospital, Heidelberg, Germany; 5 Department of Radiation Oncology, Perlmutter Cancer Center at New York, University Langone Health at Long Island, New York, NY USA; 6 German Cancer Consortium (DKTK), Partner Site, Heidelberg, Germany; 7 Department of Neurosurgery, Heidelberg University Hospital, Heidelberg, Germany; 8 Clinical Cooperation Unit Radiation Oncology, German Cancer Research Center (DKFZ), Heidelberg, Germany

**Keywords:** Proton radiotherapy, Low and intermediate grade glioma, IDH-mutation, Efficacy, Safety, Radiation-induced contrast enhancement (RICE)

## Abstract

**Purpose:**

Proton beam radiotherapy (PRT) has been demonstrated to improve neurocognitive sequelae particularly. Nevertheless, following PRT, increased rates of radiation-induced contrast enhancements (RICE) are feared. How safe and effective is PRT for IDH-mutated glioma WHO grade 2 and 3?

**Methods:**

We analyzed 194 patients diagnosed with IDH-mutated WHO grade 2 (n = 128) and WHO grade 3 (n = 66) glioma who were treated with PRT from 2010 to 2020. Serial clinical and imaging follow-up was performed for a median of 5.1 years.

**Results:**

For WHO grade 2, 61% were astrocytoma and 39% oligodendroglioma while for WHO grade 3, 55% were astrocytoma and 45% oligodendroglioma. Median dose for IDH-mutated glioma was 54 Gy(RBE) [range 50.4–60 Gy(RBE)] for WHO grade 2 and 60 Gy(RBE) [range 54–60 Gy(RBE)] for WHO grade 3. Five year overall survival was 85% in patients with WHO grade 2 and 67% in patients with WHO grade 3 tumors. Overall RICE risk was 25%, being higher in patients with WHO grade 2 (29%) versus in patients with WHO grade 3 (17%, p = 0.13). RICE risk increased independent of tumor characteristics with older age (p = 0.017). Overall RICE was symptomatic in 31% of patients with corresponding CTCAE grades as follows: 80% grade 1, 7% grade 2, 13% grade 3, and 0% grade 3 + . Overall need for RICE-directed therapy was 35%.

**Conclusion:**

These data demonstrate the effectiveness of PRT for IDH-mutated glioma WHO grade 2 and 3. The RICE risk differs with WHO grading and is higher in older patients with IDH-mutated Glioma WHO grade 2 and 3.

**Supplementary Information:**

The online version contains supplementary material available at 10.1007/s11060-022-04217-y.

## Introduction

Histopathologic and molecular characteristics dictate the biological behavior of primary brain tumors. Classification of tumor aggressiveness and ultimate prognosis has been performed by the World Health Organization (WHO) as well as the cIMPACT-NOW criteria that consider histopathology, molecular signatures, and imaging characteristics [1–3]. Diffuse gliomas are defined by growth pattern, clinical behavior and shared isocitrate dehydrogenase (IDH) genetic status. The IDH mutational status can usually be found by immunohistochemical staining but sometimes requires DNA sequencing approaches necessary to identify less common mutations [4]. In the modern era, IDH mutational status has been centrally anchored to the 5th edition of WHO classification of central nervous system tumors as it has been demonstrated to significantly influence prognosis [5]. While median survival in patients with IDH-mutated diffuse glioma is 6–10 + years, median survival in patients with IDH wildtype diffuse glioma is dramatically lower with 1–4 years [6]. Epigenetic molecular signatures, including DNA methylation status, are also carefully reviewed when classifying gliomas [7]. It is likely that the future of primary brain tumor categorization will further augment attention placed on molecular characteristics. This has been manifested as a clear shift to molecular over histopathological classification in clinical trials and modern publications [8].

Standard treatment options for IDH-mutated WHO grade 2 gliomas following resection include observation, chemotherapy and radiotherapy. Based upon RTOG 9802, the majority of patients is treated with postoperative radiation therapy and chemotherapy [9, 10]. Individual treatment depends on residual tumor following surgery, symptoms, size, risk (e.g. Pignatti criteria for low-grade glioma [11]) and tumor characteristics [9, 12–15]. In case of IDH-mutated WHO grade 3 gliomas, sequential chemoradiation following resection is the preferred treatment [6, 16–18].

Proton beam radiotherapy (PRT) has emerged as a new radiation modality option in the treatment of CNS malignancies, including gliomas, due to the superior physical dose distribution offered by proton beams relative to that seen in standard x-ray-based therapy [30]. It has been shown to decrease integral radiation dose exposure, yielding better neurocognitive outcomes [19, 20] without compromising disease control [21]. The fact that protons spare surrounding healthy tissue may also contribute to lower rates of endocrinopathy [22] and leucopenia which is an important criterion for patient chemotherapy eligibility [23]. The potential benefit of less leucopenia and therefore better chemotherapy protocol adherence is currently under evaluation for glioma WHO grade 2–3 in the GLIoProPH trial (University Hospital Essen, Germany, not yet registered on ClinicalTrials.gov) and for glioblastoma in the currently recruiting randomized prospective GRIPS trial (University Hospital Heidelberg, Germany) [24].

Despite the clear physical dose superiority given to proton therapy as a consequence of the Bragg peak, there is increasing in vitro evidence of a radiobiological effectiveness of protons beyond the current globally accepted factor of 1.1, particularly in the distal part of the Bragg peak [25]. The globally accepted factor of 1.1 was always known to be just an estimation but uncertainties in biological dose calculation were thought to be too small to be clinically relevant. But today we suspect, that these uncertainties in biological dose calculation might have a clinical impact. It might put the patient at risk of local overdose, especially at this mentioned distal part of the Bragg peak. One of the manifestations of this previously unrealized relative biological effectiveness (RBE) elevation is the risk of radiation-induced contrast enhancement (RICE), particularly when associated with chemotherapy [26–28]. In a prior publication we analyzed the cases of 227 patients (42 children and 185 adults) treated with PRT (54 Gy RBE) for low-grade-glioma WHO grade 1–2. Adults and WHO grade 2 histology was associated with an elevated risk of RICE occurrence [29]. In another prior publication including 99 patients diagnosed with RICE who were previously treated with either photon or proton therapy for World Health Organization (WHO) grade 1–3 primary gliomas, we demonstrated treatment-related factors including chemotherapy and re-irradiation characteristics were associated with an elevated risk of RICE occurrence [30]. In the subsequent present study, we investigate long-term tumor control and toxicity rates again with a particular focus on RICE in the largest homogenous cohort of patients diagnosed with IDH-mutated gliomas WHO grade 2–3 treated with PRT.

## Patients and methods

### Patient characteristics

In this study, we analyzed 194 consecutive adult patients (≥ 21 years), including 131 WHO grade 2 gliomas and 66 WHO grade 3 gliomas, all with documented IDH mutation. Patients underwent PRT between 2010 and 2020 at our institution. Patient and treatment data were extracted from the official ‘Heidelberger Institute for Radiation Oncology’ (HIRO) database and associated medical records. Survival data was obtained from the national registration office. Patients were carefully followed with routine clinical examinations and follow-up magnetic resonance imaging (MRI) as per standard institutional guidelines. For consistency and accuracy, all available follow-up MRI scans (n = 2,415) were reviewed independently by at least one radiologist and one radiation oncologist.

### Planning and treatment features

Patients were immobilized with custom thermoplastic masks and treatment planning simulation scans were obtained, including computed tomography (CT) as well as cranial MRI (cMRI). Gross tumor volume (GTV) included the macroscopic tumor and/or resection cavity. For the clinical target volume (CTV), a 1.5–2 cm margin was added to account for suspected microscopic tumor spread while respecting anatomic boundaries. An isotropic margin of 3 mm was used for creation of the planning target volume (PTV) to account for geometric uncertainties and physical inaccuracies of the beam.

Treatment planning followed the principle of as low as reasonably achievable (ALARA) and was according to the constraints of ICRU report 50 and 62 as well as normal tissue constraints according to QUANTEC and Emami et al. [31]. Two to three treatment beams using active raster-scanning technique were used under daily image guidance with orthogonal x-ray imaging. The final proton dose was scaled with a constant relative biological effectiveness (RBE) factor of 1.1. Treatment was delivered over 5–6 fractions per week. According to the current guidelines, WHO grade 2 gliomas were treated with a dose of 50.4–54 Gy/Gy(RBE) in 1.8–2.0 Gy/Gy(RBE) per fraction and WHO grade 3 gliomas with 54–60 Gy/Gy(RBE) in 1.8–2.0 Gy/Gy(RBE) per fraction [32]. If the tumor was close to relevant organs at risk (e.g. optical system, brainstem), irradiation dose was reduced locally and minor dose dips may have occurred within the PTV to achieve safe OAR dose constraints. All cases adhered to ASTRO guidelines for radiation therapy indications, advanced radiation therapy technique utilization, and clinical management of adverse effects [33].

### Endpoint definition

Primary endpoints were oncologic effectiveness and toxicity of PRT. Intracranial control was evaluated using the Response Assessment in Neuro-Oncology (RANO) criteria [34] and divided into no progression (classified by complete/partial response, or stable disease) and progression. If disease progression was suspected, this was confirmed with additional follow-up cMRIs to distinguish it from transient post-treatment edema versus RICE. Toxicity was graded following the National Cancers Institute’s Common Terminology Criteria for Adverse Events (CTCAE) (version 4.03). The assessment of mild and expected toxicities such as fatigue can be very challenging to reflect correctly in a retrospective analysis, thus we focused on (RICE) and severe (CTCAE grade ≥ 3) other toxicities for this analysis.

**Fig. 1:**
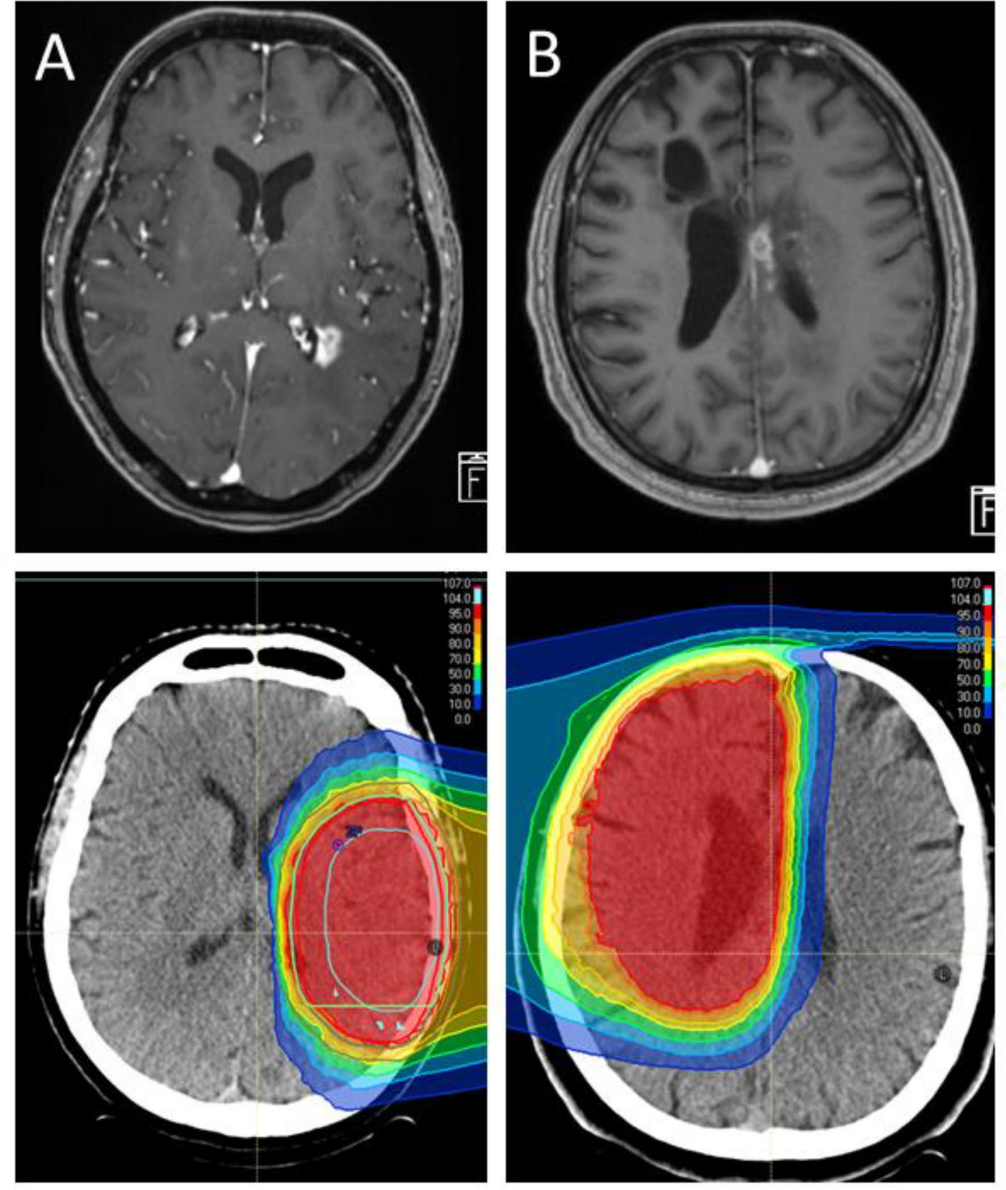
Two typical examples of periventricular radiation-induced contrast enhancements (RICE) next to the corresponding radiotherapy plan. The patient in example case A is a 53 year-old female with fibrillary astrocytoma WHO grade 2 that underwent definitive radiation therapy [54 Gy(RBE) in 27 fractions protons]. The patient in example case B is a 50 year-old male with anaplastic astrocytoma WHO grade 3 that underwent adjuvant chemoradiation (60 Gy(RBE) in 30 fractions protons; temozolomide). Colored isodoses demonstrate relative dose [%] of prescription dose [Gy]

We defined RICE as new post-treatment contrast enhancement on cMRI in surrounding brain tissue within the 80% isodose line analogous to RANO criteria [34] during the follow-up period. Two examples of RICE are shown in Fig. 1.

Cases were evaluated using all available cMRIs, radiation treatment plans, and medical records that reflect time course and concurrent therapies. Each retrospectively suspected RICE case was (re)presented to the multidisciplinary tumor board. Following this approach, we were able to minimize the likelihood of misinterpreting RICE as tumor progression in our analysis, as previously published for a preceding project [30]. Patients with small RICE or stable RICE in noncritical, symptom-free locations underwent only follow-up visits and did not receive RICE-directed therapy. If RICE was significant, progressive, in critical/eloquent locations and/or symptomatic, patients received RICE-directed therapy. In general, RICE-directed therapy was started with dexamethasone. Bevacizumab was added if dexamethasone did not sufficiently improve symptoms. If symptoms were severe, bevacizumab was started immediately. This approach was chosen according to the DEGRO practical guideline for Central Nervous System Radiation Necrosis Part I: Classification and a multi-step approach for Diagnosis [35] and DEGRO practical guideline for Central Nervous System Radiation Necrosis Part II: Treatment of the German Society for Radiation Oncology [36].

Secondary endpoints included overall survival (OS), progression-free survival (PFS), RICE-free survival, and CTCAE version 4.0 defined toxicity. To verify unresolved side effects according to the National Cancers Institute CTCAE ≥ °1 (version 4.03), medical records and imaging reports were analyzed. The first regular follow-up cMRI was performed 4–8 weeks after the end of radiotherapy. If no unexpected lesions were found, the next cMRI was performed in time intervals of 8–12 weeks.

### Statistical analysis

Descriptive statistics for baseline variables (Tables 1 and 2) and follow-up data (Table 3) include means (standard deviation) and/or medians (IQR and range, as appropriate) for continuous variables, and absolute and relative frequencies for categorical variables. For time-based endpoints, Kaplan–Meier estimates were calculated. Strata were compared using the log-rank test. To identify prognostic factors on PFS and OS during the follow-up period, Cox proportional hazard models were used. Results are presented in terms of Hazard Ratio (HR), corresponding confidence interval (CI), and p-value. Because this is an exploratory analysis, p-values are descriptive in nature. A p-value of < 0.05 was considered statistically significant. Statistical analyses were performed with R software Version 4.0.2 (r-project.org). 

## Results

The median age of patients at the start of radiotherapy was 39.4 years (range: 21.1–76.3 years) in the overall cohort. Slightly more men than women were included (57% vs. 43%). The histological subtype was predominately astrocytoma in (59%) with the remainder oligodendroglioma. Tumors were classified as WHO grade 2 in 66% and WHO grade 3 in 34%. Detailed patient characteristics are shown in Table 1.

**Table 1:** Baseline characteristics

	Overall cohort	WHO grade 2 tumors	WHO grade 3 tumors
Gender	n = 194 [%]	n = 128 [%]	n = 66 [%]
Female	84 [42.8%]	52 [40.6%]	31 [47.0%]
Male	113 [57.2%]	76 [59.4%]	35 [53.0%]
Age at primary diagnosis n = 194 [%]		n = 128 [%]	n = 66 [%]
Median	36.4	35.5	36.5
Minimum–Maximum	15.3–76	15.3–76	18.6–66.7
Karnofsky performance status n = 177 [%]		n = 114 [%]	n = 63 [%]
≤ 70	16 [10.4%]	13 [11.4%]	5 [7.9%]
80	22 [12.5%]	12 [10.5%]	10 [15.9%]
90	78 [44.3%]	50 [43.9%]	29 [46.0%]
100	58 [32.8%]	39 [34.2%]	19 [30.2%]
Diagnosis	n = 194 [%]	n = 128 [%]	n = 66 [%]
Astrocytoma	116 [58.9%]	76 [60.9%]	36 [54.6%]
Oligodendroglioma (1p/19q LOH deletion)	81 [41.1%]	50 [39.1%]	30 [45.4%]
MGMT promotor methylated n = 103 [%]		n = 59 [%]	n = 34 [%]
No	16 [15.5%]	10 [16.9%]	6 [13.6%]
Yes	87 [84.5%]	49 [83.1%]	38 [86.4%]

If not otherwise visible, absolute and relative frequencies were shown. Relative frequencies are based on the available data and exclude missings

Initial treatment included surgical resection, partial or complete, in 67% of patients. The remaining 33% of patients underwent biopsy only. Chemotherapy was used as a component of treatment in the vast majority of both WHO grade 2 (82%) and 3 (98%) tumors. Detailed treatment characteristics are shown in Table 2.

**Table 2:** Treatment characteristics

	Overall cohort n = 194 [%]	WHO 2 tumors n = 128 [%]	WHO 3 tumors n = 66 [%]
Past surgery			
Biopsy	63 [32.5%]	52 [40.6%]	11 [16.7%]
Subtotal resection	57 [29.4%]	38 [29.7%]	19 [28.8%]
Total resection	74 [38.1%]	38 [29.7%]	36 [54.6%]
Chemotherapy			
None	24 [12.4%]	23 [18.0%]	1 [1.5%]
PCV	50 [25.8%]	35 [27.3%]	15 [22.7%]
Temozolomide	109 [56.2%]	60 [46.9%]	49 [74.2%]
Other	11 [5.7%]	10 [7.8%]	1 [1.5%]
Time diagnosis until radiotherapy start (years)			
Median	1.6	2.0	0.7
Minimum–maximum	0.1–20.6	0.1–20.6	0.1–14.7
Total dose (Gray RBE)			
Median	54.0	54.0	60
Minimum–maximum	50.4–60.0	50.4–60.0	54.0–60.0
Dose per fraction (Gray RBE)			
1.8 Gy	86 [44.3%]	60 [46.9%]	26 [39.4%]
2 Gy	108 [55.7%]	68 [53.1%]	40 [60.6%]

If not otherwise visible, absolute and relative frequencies were shown. Relative frequencies are based on the available data and exclude missings

*Gy RBE *Gray Relative Biological Effectiveness; *PCV* Procarbazine/Lomustine/Vincristine regimen

The median follow-up period was 5.1 years (range, 7 months to 11 years) and was similar in the subgroups with WHO 2 and WHO 3 tumors, respectively. The analysis included in total 2403 cMRIs that were reviewed independently by two specialists. MRI-graphical follow-up was similar between the subgroups. As expected, MRI response to radiotherapy according to RANO criteria differed between the subgroups and preferred the subgroup with WHO 2 tumors. Follow-up characteristics can be found in Table 3.

**Table 3:** Follow-up characteristics

	Overall cohort n = 194 [%]	WHO grade 2 tumors n = 128 [%]	WHO grade 3 tumors n = 66 [%]
Total follow-up period (years)			
Median	5.1	5.0	5.3
Minimum–maximum	0.6–11.3	0.6–11.3	0.6–9.9
Vital state at the end of the follow-up period			
Dead	44 [22.7%]	21 [16.4%]	23 [34.9%]
Alive	150 [77.3%]	107 [83.6%]	43 [65.1%]
Number of follow-up MRIs per patient			
Median	11	10	12
Minimum–maximum	1–40	1–38	1–40
MRI response to radiotherapy in the overall cohort			
Regression or stable disease	116 [59.8%]	87 [68.0%]	29 [43.9%]
Progressive disease	78 [40.2%]	41 [31.0%]	37 [56.1%]

If not otherwise visible, absolute and relative frequencies were shown. Relative frequencies are based on the available data in each subgroup and exclude missings

**Fig. 2:**
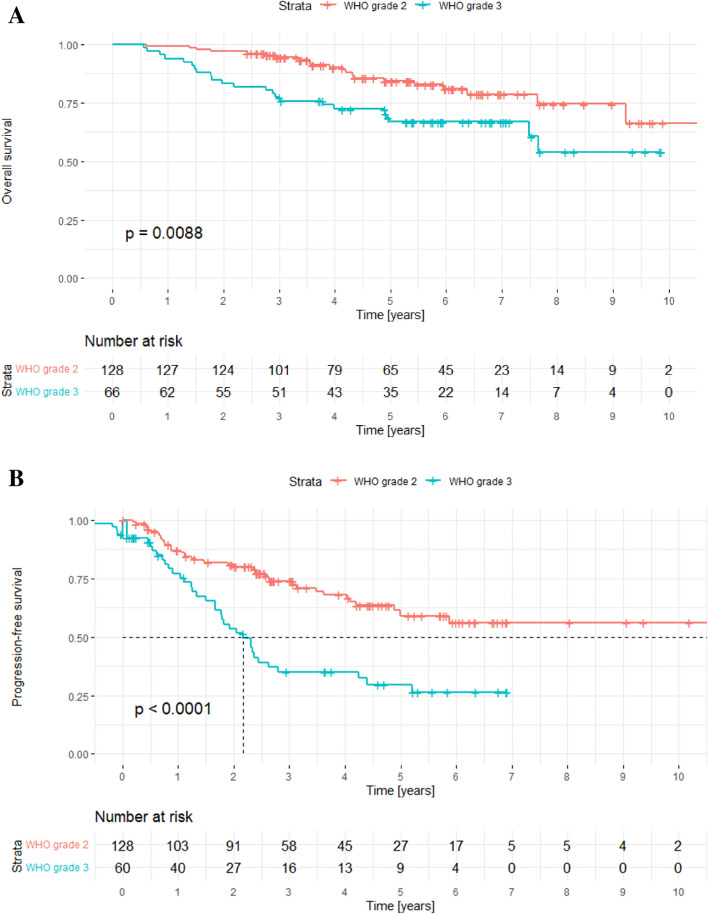
Overall survival stratified by WHO grade (**A**) and progression-free survival stratified by WHO grade (**B**)

At the time of analysis, in the overall cohort, 150 patients (77%) were alive and 44 patients (23%) had died. Median overall survival was not reached during the follow-up period for both patients with WHO grade 2 and 3 tumors. Five year overall survival after PRT was 85% in patients with WHO grade 2 and 67% in patients with grade 3 IDH-mutated gliomas (p = 0.0088, see also Fig. 2A).

As previously described in the methods section, PFS was carefully assessed because at first appearance, RICE may be difficult to distinguish from tumor progression. Overall, 25% of patients (n = 49) developed RICE, thus the interpretation of treatment response may be difficult in this subgroup. Therefore, PFS was recalculated only in the subgroup without RICE (n = 145) to exclude RICE misinterpretation bias (Supplementary Fig. 1). The 5 year PFS was 30% in WHO grade 3 IDH-mutated gliomas and 60% in WHO grade 2 IDH-mutated gliomas (p < 0.0001). PFS in the overall cohort and in the subcohort without RICE were exactly the same in our analysis (Fig. 2B and Supplementary Fig. 1). Thus, RICE occurrence neither influenced OS (p = 0.15) nor PFS (p = 0.67). These results also demonstrate data quality assurance with the above-mentioned diagnostic approach as it excludes RICE misinterpretation bias in our data.

Follow-up MRIs in the overall cohort revealed evidence of RICE after PRT in 49 cases (25%). After a median time of 16 months (range: 2–41 months) following PRT, RICE was observed and was more common in WHO grade 2 (29%) versus grade 3 (17%) IDH-mutated gliomas, but the difference did not reach the threshold for significance (p = 0.11). Analysis for factors influencing time latency between radiotherapy and first RICE occurrence was conducted. We analyzed patient-related, tumor-related and treatment-related factors in multivariable linear regression modelling and astrocytoma histopathology (vs. oligodendroglioma histopathology; p = 0.047), Bragg peak/steep dose falloff (p = 0.037) and re-irradiation (p = 0.004) in case of tumor recurrence to go along with a shorter time latency to first RICE occurrence, independent of age, sex, Karnofsky status, WHO grading, total dose, fraction dose, resection, chemotherapy and dose at the periventricular zone.

**Fig. 3:**
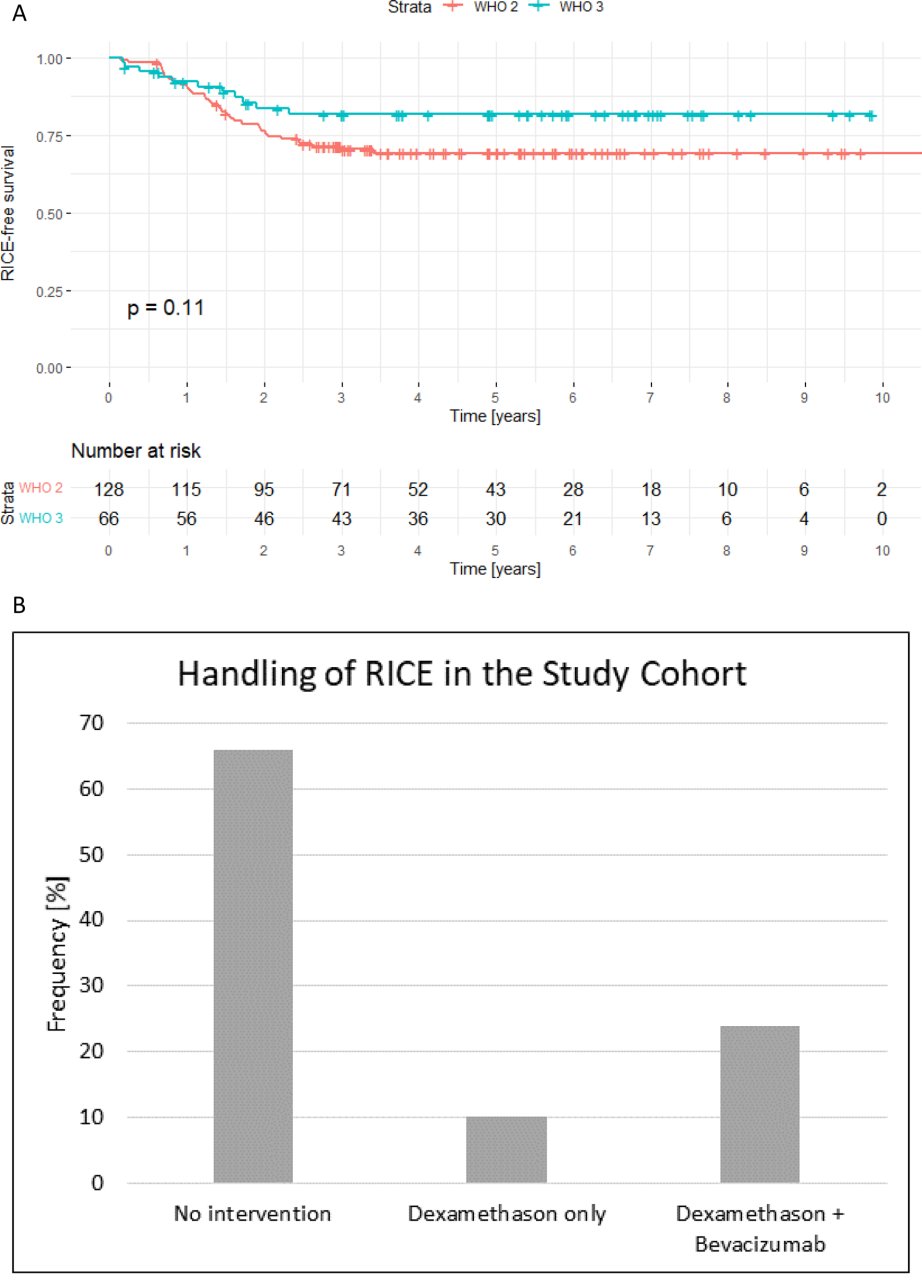
**A** Radiation-induced contrast enhancements (RICE)-free survival stratified by WHO grade. RICE did occur within the first 2 years following radiation therapy in most cases. As re-irradiation could influence time latency to RICE occurrence, the 21 patients that received re-irradiation were censored at the timepoint of re-irradiation. This was in median after 5.4 years following first irradiation. **B** Radiation-induced contrast enhancements (RICE) in the study cohort were handled with Bevacizumab plus Dexamethasone, Dexamethasone only and no RICE-directed therapy, depending on RICE size, localization and symptoms

RICE-free survival stratified by WHO classification is shown in Fig. 3A. Independent of histologic subtype (oligodendroglioma vs. astrocytoma) and WHO grading, RICE risk increased with higher age in multivariable Cox regression analysis (p = 0.017). Clinical symptoms were observed in 15 patients (31% of RICE cases). If symptoms were present, they were generally mild (CTCAE grade 1). The resulting CTCAE toxicity distribution was grade 0 (n = 34; 69%), grade 1 (n = 12, 25%), grade 2 (n = 1; 2%), and grade 3 (n = 2; 4%). No RICE cases were classified as CTCAE > 3. Of all RICE cases, on a minority, 35% (n = 17), received RICE-directed treatment. In general, the criteria for RICE-directed therapy, including corticosteroids alone or corticosteroids plus bevacizumab, were clinical symptoms (e.g., headache, dizziness, visual disturbances, focal neurological deficits), radiological progression, or critical localization (e.g., optic system, brainstem, or eloquent areas) independent of clinical symptoms. Of note, the anti-vascular endothelial growth factor (VEGF) antibody bevacizumab was added in severe or corticosteroid-resistant cases. RICE-directed therapy, such as corticosteroids and bevacizumab for more severe or symptomatic RICE, led to radiological regression of RICE in 53% (n = 9) of cases with regression of symptoms in 29% (n = 5) of cases. Our study cohort indicated an overall tumor recurrence risk of 40% (n = 78) of patients. In case of a tumor recurrence, 27% (n = 21) of patients underwent re-irradiation and 88% (n = 69) received re-chemotherapy which also influenced RICE, if present: A tumor-specific therapy including chemotherapy or re-irradiation led to a RICE size progression in 86% (n = 59) and 92% (n = 19) of cases, respectively and RICE symptom progression in 57% (n = 39) and 65% (n = 14) of cases, respectively. After the diagnosis of RICE, in 65% (n = 32) no intervention was required. These cases were typically small in volume (median of 2 mL) and less symptomatic. After a median of 7.2 months (range 3–33 months) following no intervention, RICE size regression was observed in 67% (n = 21) of these cases without intervention and RICE symptom regression was observed in 27% (n = 9) of cases (all were low grade toxicities CTCAE °I-II). The remaining 6% (n = 2) of cases without intervention demonstrated with stable RICE at the end of the follow-up period. Response to RICE treatment was higher in WHO grade 2 tumors than WHO grade 3 tumors. Presence of RICE did not influence overall survival (p = 0.17; see also Supplementary Fig. 2). Details on RICE can also be found in Table 4 while details on RICE management are shown in Fig. 3B.

**Table 4:** Treatment toxicity: Radiation-induced contrast enhancements (RICE)

	Overall cohort n = 194 [%]	WHO grade 2 tumors n = 128 [%]	WHO grade 3 tumors n = 66 [%]
RICE occurrence			
Yes	49 [25.3]	38 [29.7%]	11 [16.7%]
No	145 [74.7]	90 [70.3%]	55 [83.3%]
Time from radiotherapy completion to first occurrence of RICE (months)			
Median	15.8	15.8	13.7
Minimum–maximum	1.7–40.9	1.7–40.9	2.1–27.8
RICE CTCAE grading	n = 49 [%]	n = 38 [%]	n = 66 [%]
CTCAE grade 0	34 [69.4%]	24 [63.2%]	10 [90.9%]
CTCAE grade 1	12 [24.5%]	12 [31.6%]	0 [0%]
CTCAE grade 2	1 [2.0%]	1 [2.6%]	0 [0%]
CTCAE grade 3	2 [4.1%]	1 [2.6%]	1 [9.1%]
CTCAE grade > 3	0 [0%]	0 [0%]	0 [0%]
RICE treatment details	n = 49 [%]	n = 38 [%]	n = 66 [%]
No treatment needed	32 [65.3%]	26 [68.4%]	6 [54.6%]
Treatment needed	17 [34.7%]	13 [31.6%]	5 [45.4%]
Steroids only	5 [10.2%]	4 [10.5%]	1 [9.0%]
Bevacizumab (anti-VEGF antibody)	12 [24.5%]	8 [21.1%]	4 [36.4%]
If RICE treatment, response	n = 17 [%]	n = 13 [%]	n = 5 [%]
Any improvement	10 [58.8%]	8 [61.5%]	2 [40.0%]
Symptom improvement	5 [29.4%]	3 [23.1%]	2 [40.0%]
Radiological improvement	9 [52.9%]	7 [53.8%]	2 [40.0%]

*RICE* Radiation-induced contrast enhancement; *CTCAE* Common Terminology Criteria for Adverse Events

Other radiation-induced toxicities were classified as low-grade and were mostly self-limiting. Typical radiation-induced side effects included fatigue, transient headache, erythema, focal alopecia, and dysgeusia. They were all classified as CTCAE < 3, and were mainly observed in the early post-irradiation period. No CTCAE grade ≥ 3 toxicities were observed.

## Discussion

Herein we report the largest cohort to date with long-term clinical and radiological outcomes in a population of 194 adult patients diagnosed with IDH-mutated WHO grade 2 and 3 gliomas treated with PRT. With a median follow-up of 5.1 years, tumor regression or stability was observed in 60% for WHO grade 2 and 30% for WHO grade 3. The estimated 5 year overall survival was 85% for patients with WHO grade 2 and 67% for patients with grade 3 IDH-mutated gliomas. Overall RICE risk was 25% and depended on WHO grading and age.

Although direct efficacy comparisons between our data and historical cohorts after photon radiotherapy are difficult due to differences in tumor-, patient-, and treatment-related factors, it is fair to say that delivery of modern PRT in this fashion is effective in both WHO grade 2 and 3 IDH-mutated gliomas based on our analysis. The EORTC 22845 trial on low-grade glioma reported a 5 year overall survival rate of 68.4% in the photon radiotherapy group, though in contrast to our trial all patients underwent resection [37]. The EORTC 22033–26033 trial demonstrated that primary photon radiotherapy versus primary temozolomide was associated with an improved PFS for WHO grade 2 gliomas with a 5 year PFS of 40% versus 29% [15]. The best results were observed in the RTOG 9802 in which sequential photon radiotherapy and chemotherapy in high risk patients (i.e. age ≥ 40 years and/or subtotal resection) achieved dramatic improvements in median overall survival (13.3 vs. 7.8 years) [9, 10]. Turning to the results of particle therapy, in the low-grade glioma population, 5 year overall survival has been reported at 84%, which is nearly identical to the results in our subcohort of WHO grade 2 tumors (85%) [38]. Remarkably, the 5 year PFS following PRT in LGG for adults was historically estimated as 40%, which is notably lower than that seen in our cohort measured at 60% [38]. As this difference does only affect PFS but not OS, it could be hypothesized that in historical clinical trials RICE were sometimes misinterpreted as tumor progression. This hypothesis is supported by in-house data that demonstrated a high misinterpretation risk on the one hand and RICE not influencing OS on the other hand [30].

Diving deeper into the WHO grade 3 glioma literature demonstrates that chemotherapy choice should be made based on histopathology as this significantly influences prognosis [6, 17, 39] In this analysis, most of the patients were treated with a sequential chemoradiation using temozolomide. The CATNON trial reported the subgroup of IDH-mutated WHO grade 3 gliomas had a median OS of 78–117 months, depending of temozolomide timing. In the CATNON subgroup of patients with IDH-mutated WHO grade 3 gliomas, 5 year overall survival ranged between 62 and 82%, depending on characteristics of temozolomide therapy [6]. In our real-world cohort, where chemotherapy eligibility, agents and timepoints were inhomogeneous due consideration of individual patient factors, 5 year overall survival in the subgroup of patients with WHO grade 3 tumors was 67%, comparable to the CATNON data.

In our overall cohort, 25% of patients developed RICE, which was observed after a median of 15 months after PRT (Fig. 2B and Table 4). The rate of RICE observed in the present study is considerable, but it should be noted that screening and assessment for RICE was frequent and rigorous. The RICE rate is consistent with the adult low-grade glioma cohort treated with PRT we previously published [29]. This supports the relevance of RICE as a differential diagnosis even many months or years following radiotherapy as RICE misinterpretation for tumor progression is a frequent problem, with misinterpretation rates of up to 39% at initial contrast enhancement [30]. Prior publications demonstrated RICE risk 6 months following radiotherapy does not differ between photons and protons, however the timepoint of first RICE occurrence may differ. The RICE in oligodendroglioma patients occurs earlier after proton than photon radiotherapy, in contrast time to RICE occurrence in astrocytoma was independent of radiotherapy modality (protons vs. photons) [40]. Importantly, neither PFS nor OS was affected by the occurrence of RICE, which is feared and therefore frequently discussed in the community. These data demonstrate after analysis of 194 adult patients and 2403 contrast-enhanced cMRIs, RICE do not influence prognosis of patients. In addition, we identified two factors that went hand in hand with a higher risk of developing RICE: [1] older age, and [2] WHO 2 grading, with the former but not the latter independently meeting the significance threshold. We previously found similar associations for low-grade gliomas in children and adults [29].

In the present study, the RICE rate for IDH-mutated glioma was two-fold higher in WHO grade 2 (29%) compared to grade 3 (17%, difference not statistically significant). Historical data has reported wide ranging rates of overt RICE in pediatric patients treated with PRT or photon radiotherapy for low-grade glioma ranging from 1 to 34%, with similar rates for both photons and protons [21, 27, 41]. In adult patients, rates of RICE following photon radiotherapy for glioma are seemingly higher (30–34%) [27, 42]. In adults with low-grade glioma, RICE was more frequent following PRT compared to photon IMRT [27]. Clearly, the pathology of the primary brain tumor influences the surrounding tumor microenvironment. Prior low-grade glioma surgical series have elucidated differences in surrounding tumor vasculature of pediatric pilocytic astrocytomas (i.e. degenerative vascular changes) and adult astrocytomas (i.e. microvascular proliferation) [43–45]. These vascular pathological differences may give hints for the age-dependence of RICE risk found in our analysis. Moreover, physiologic vasculature is well known to be age-dependent and age is one of the most important risk factors for macro- and- micro-vascular changes. There is likely an interplay of an increased age-dependent tissue radiosensitivity with the elevated radiobiological characteristics of proton therapy in the manifestation of this treatment-related complication. Future research should explore this topic to better understand patients’ individual risk, so that treatment and follow-up measures can be adapted accordingly.

RICE lesions can be very difficult to distinguish from disease progression. As a result, salvage therapies such as chemotherapy, re-resection, or re-irradiation may be inadvertently pursued following RICE diagnosis despite lack of progression. Additional tumor-directed therapy can further aggravate the blood brain barrier disruptions present in RICE leading to progressive RICE lesions in up to 86% (with chemotherapy) or 92% (with re-irradiation) [30]. In effort to better categorize RICE versus tumor progression, metrics are being developed at our institution which consider patient-related, tumor-related, and treatment-related (e.g. LET) factors that influence the risk of RICE development [46, 47]. Our understanding of the biologic effectiveness of PRT in tissues requires improvement. Robust radiobiological models and predictive biomarkers for individual PRT plans are lacking and should be areas of future research.

A significant challenge in collecting data from a long-term data set (2010–2020) is understanding the treatment effect in the context of our rapidly changing understanding of primary brain tumors from a molecular standpoint. For this analysis, we only included WHO grade 2–3 glioma patients with evidence of an IDH mutation. This also reduced bias caused by the changes in WHO classifications during 2010–2020. Our analysis is lacking a direct photon comparison arm, but compares data to historical prospective cohorts. From a radiation oncologists´ standpoint, PRT is not a broadly available radiotherapy technique. Many patients self-select for such treatment and travel to proton centers which may lead to selection bias and reduce the generalizability to the broader glioma patient population. Moreover, given the international presence of our institution, some patients who were treated were lost to follow up once they returned home. Nevertheless, a median follow-up period of 5.1 years in a diffuse glioma cohort of 197 patients augments the strength of our analysis and offers a detailed look at the efficacy and toxicity of PRT in the management of IDH-mutated WHO 2 and WHO 3 low-grade gliomas.

## Conclusion

We demonstrate effectiveness of PRT for adult patients with both IDH-mutated WHO grade 2 and 3 gliomas with a 5 year overall survival of 85% and 67% respectively, which is therefore comparable to other cohorts treated with radiotherapy and does not demonstrate any effectiveness differences between proton and photon radiotherapy based on historical comparisons. The RICE risk differs with tumor grading and was 29% in WHO grade 2 and 17% grade 3 tumors. One third of RICE cases received a RICE-directed therapy due to RICE symptoms, size or localization, which was also effective and relieved clinical symptoms and radiological changes in 29% and 53%, respectively. Moreover, RICE did not affect overall survival or PFS. These data support the safety and efficacy of PRT for patients with IDH-mutated WHO grade 2–3 gliomas.

## Supplementary Information

Below is the link to the electronic supplementary material.

Supplementary file1 (PNG 12 kb)

Supplementary file2 (PNG 11 kb)

## Data Availability

The datasets generated for this study will not be made publicly available since national legislation and the terms of study ethics approval do not allow dataset sharing outside of the institutions participating in the analysis.
