# Supplementary figures and images for: Analysis of safety and efficacy of proton radiotherapy for IDH-mutated glioma WHO grade 2 and 3
Source: J Neurooncol. 2023 Jan 4;162(3):489–501. doi: 10.1007/s11060-022-04217-y (PMC10227167; doi:10.1007/s11060-022-04217-y)

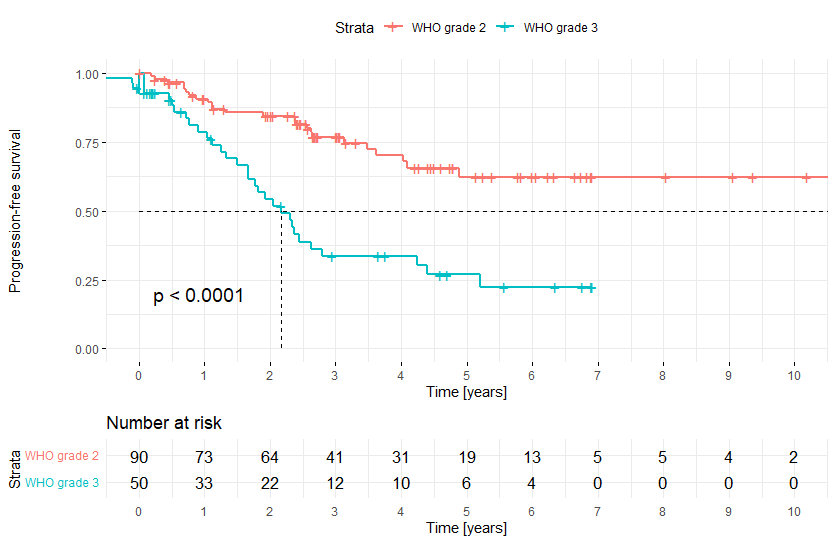

Supplement: Supplementary file 1 — Supplementary file1 (PNG 12 kb) [file 11060_2022_4217_MOESM1_ESM.png]

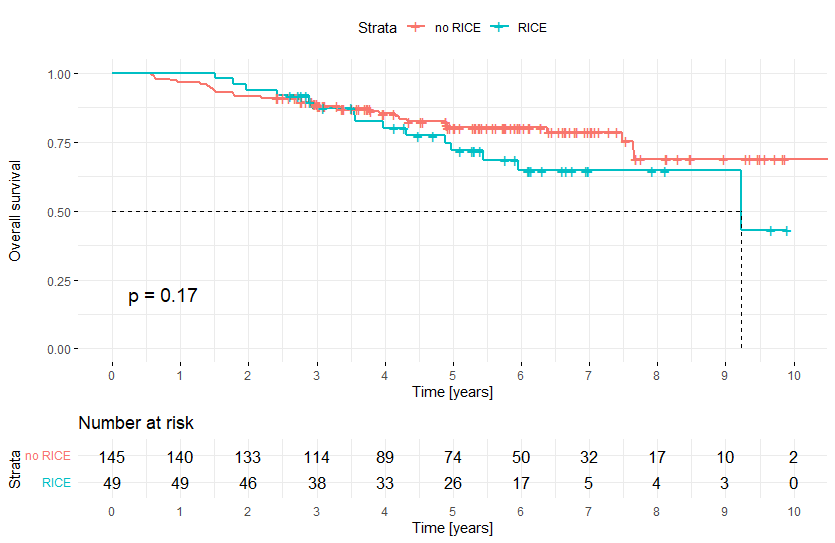

Supplement: Supplementary file 2 — Supplementary file2 (PNG 11 kb) [file 11060_2022_4217_MOESM2_ESM.png]
